# *Candida albicans* Infection Disrupts the Metabolism of Vaginal Epithelial Cells and Inhibits Cellular Glycolysis

**DOI:** 10.3390/microorganisms12020292

**Published:** 2024-01-30

**Authors:** Yanni Zhao, Pengjiao Wang, Xiaodong Sun, Mei Zhao, Yixuan Chen, Xiuli Gao

**Affiliations:** 1 State Key Laboratory of Functions and Applications of Medicinal Plants, School of Pharmacy, Guizhou Medical University, Guiyang 550031, China; 2 Microbiology and Biochemical Pharmaceutical Engineering Research Center, Guizhou Provincial Department of Education, Guizhou Medical University, Guiyang 550031, China

**Keywords:** *Candida albicans*, vaginal epithelial cells, vulvovaginal candidiasis, metabolomics, glycolysis

## Abstract

Vulvovaginal candidiasis (VVC) is a common gynecologic disorder caused by fungal infections of the vaginal mucosa, with the most common pathogen being *Candida albicans* (*C. albicans*). Exploring metabolite changes in the disease process facilitates further discovery of targets for disease treatment. However, studies on the metabolic changes caused by *C. albicans* are still lacking. In this study, we used *C. albicans*-infected vaginal epithelial cells to construct an in vitro model of VVC, analyzed the metabolites by UHPLC-Q-Exactive MS, and screened the potential metabolites based on metabolomics. The results showed that *C. albicans* infection resulted in significant up-regulation of D-arabitol, palmitic acid, adenosine, etc.; significant down-regulation of lactic acid, nicotinamide (NAM), nicotinate (NA), etc.; and disruption of amino acid metabolism, and that these significantly altered metabolites might be potential therapeutic targets of VVC. Further experiments showed that *C. albicans* infection led to a decrease in glycolytic enzymes in damaged cells, inhibiting glycolysis and leading to significant alterations in glycolytic metabolites. The present study explored the potential metabolites of VVC induced by *C. albicans* infection based on metabolomics and verified the inhibitory effect of *C. albicans* on vaginal epithelial cell glycolysis, which is valuable for the diagnosis and treatment of VVC.

## 1. Introduction

VVC is the second most common inflammatory disease of the vagina after bacterial vaginitis. It often presents clinically with symptoms such as vulvar itching, vaginal pain, and increased discharge. According to foreign epidemiological studies, about 75% of women of reproductive age have the disease once in their lifetime, and about 50% have it twice or more [1], which seriously affects women’s health and quality of life. The main pathogens of VVC are *Candida* spp., including *Candida albicans*, *Candida smoothus*, and *Candida tropicalis* [2]. *C. albicans* is the most common pathogen in women with VVC and can be isolated in 75% to 90% of cases [3].

Because of its high prevalence and recurrence rate, timely diagnosis of VVC is particularly important for the control of disease progression. We can determine whether a patient is infected with *C. albicans* by observing the clinical symptoms or performing fungal cultures [4]. However, these methods often do not allow us to make a quick and accurate judgment. In recent years, metabolomics has been widely used to study key metabolites of diseases, and it is an effective tool for diagnosis and treatment of diseases [5]. However, studies on disease metabolites for VVC induced by *C. albicans* infection are still lacking. In this study, we infected the human vaginal epithelial cells, which are commonly used for in vitro vaginitis studies, with *C. albicans*, the most common pathogen causing VVC, to construct an in vitro model of VVC, analyzed the metabolites using UHPLC-Q-Exactive MS, and explored the differential metabolites based on metabolomics.

*C. albicans* is a commensal and opportunistic pathogen that can cause various diseases when the body’s immunity is out of balance, most commonly superficial infections of the vaginal mucosa, esophagus, and skin [6]. The pathogenic mechanisms reported for *C. albicans* include mainly the changes in *C. albicans* itself during infection, as well as the effects on epithelial cells. *C. albicans* adhering to the vaginal mucosa changes its morphology from a free yeast state to an invasive hyphae state [7], facilitating its further colonization and adhesion to cells and penetrating the barrier into the deeper tissues. The invasion of epithelial cells by *C. albicans* can affect the cellular immune response by activating signaling pathways such as TLR and MAPK [8]. In addition, *C. albicans* secretes virulence factors that enhance invasion and infection of cells, including cell wall adhesins (ALS, EPA, HWP1), secreted aspartyl proteinases (Saps), candidalysin, and phospholipases [9]. Saps and candidalysin are considered the most important virulence factors. Saps promote the adhesion of *C. albicans* and facilitate hyphae penetration into tissues by hydrolyzing host histones. The interaction of high concentrations of candidalysin with cell membranes can cause pore-like structures in the cell membrane, leading to cell membrane damage [10,11].

In addition, previous studies have shown that during *C. albicans* infection of cells, the cytoarchitecture of vaginal epithelial cells is damaged by *C. albicans* hyphae and virulence factors [12], and it can be inferred that this would lead to a massive release of the cellular damage marker lactate dehydrogenase (LDH) [13]. A large loss of LDH, an important enzyme in glycolysis, may result in the inhibition of glycolysis in vaginal epithelial cells. Glycolysis is an essential metabolic pathway for the production of ATP and the activation of immune cells [14], and its reaction intermediates are involved in various biosynthetic pathways, which are essential for maintaining normal cellular life activities [15,16]. However, there is insufficient evidence that *C. albicans* affects the glycolysis of vaginal epithelial cells, and there is a lack of studies exploring the mechanism of action of *C. albicans* on vaginal epithelial cells from the metabolomic perspective. Therefore, we examined the metabolic effects of *C. albicans* on vaginal epithelial cells by detecting changes in cellular metabolites, key metabolites, and enzymes involved in glycolysis and observed whether cellular glycolysis levels were suppressed in infection, which could be valuable in further exploring the pathogenesis of *C. albicans*.

## 2. Materials and Methods

### 2.1. Cell Culture

The human vaginal epithelial cells (VK2/E6E7, CRL-2616, ATCC, Manassas, VA, USA) were cultured in Dulbecco’s modified Eagle medium (DMEM, PM150223, Procell Life Sciences & Technology, Wuhan, China) added with 1% penicillin–streptomycin–gentamicin mixed solution (Solarbio, Beijing, China) and 10% FBS (VivaCell Biosciences, Shanghai, China) and maintained at 37 °C in a humidified atmosphere with 5% CO_2_. *C. albicans* (BNCC186382, BeNa Culture Collection, Beijing, China) was cultured 24 h aerobically in liquid YM medium (0.3% *w*/*v* yeast extract, 0.3% *w*/*v* malt extract, 1% *w*/*v* dextrose, and 0.5% *w*/*v* peptone) at 37 °C, collected and resuspended in PBS, and stored at 4 °C for use.

### 2.2. Groups and Treatment

Three groups were set up for this experiment: control group, model group, and amphotericin B (AmB) group. *C. albicans* cells were cocultured with vaginal epithelial cells for 24 h of infection to simulate an in vitro model of VVC [6]. The cells were seeded in cell culture flasks and incubated at 37 °C. After 24 h, the medium was discarded, and DMEM was added to the control group; DMEM containing *C. albicans* was added to the model and AmB groups. After 24 h, the medium was discarded, and DMEM was added to the control and model groups; DMEM containing 0.5 μg/mL AmB was added to the AmB group. After 24 h, the supernatant was collected and stored at −80 °C for glucose, pyruvic acid, lactic acid, LDH, and inflammatory factors assay, and the cultures were washed with PBS and stored at −80 °C for metabolism analysis.

### 2.3. Metabolomics Analysis

The cultures were added with 1mL 80% methanol aqueous solution, ground in the freeze grinder, and centrifuged at 4 °C, 14,000 rpm for 15 min. The supernatant was evaporated with nitrogen and re-dissolved by adding 50% methanol aqueous solution to precipitate the protein fully, and then centrifuged at 4 °C, 14,000 rpm for 15 min, and collected the supernatant for UHPLC-Q-Exactive Plus Orbitrap MS (Thermo Fisher Scientific, Waltham, MA, USA) analysis.

UHPLC-Q-Exactive Plus Orbitrap MS equipped with Thermo Hypersil GOLD column (150 × 2.1 mm^2^, 1.9 μm, 40 °C) was used to detect the samples (injection volume: 4 μL). The flow rate was 0.300 mL·min^−1^. The 19 min gradient (equate A: H_2_O, 0.1% formic acid; equate B: acetonitrile, 0.1% formic acid) was set as follows: 2% B at 0–2.5 min; 2–40% B at 2.5–5 min; 40–100% B at 5–12 min; 100% B at 12–16 min; 100–2% B at 16–16.1 min; 2% B at 16.1–19 min. The mass spectrometry conditions were set as follows: ion source, HESI; ion spray voltage, 3500/2800V (+/−); capillary temperature, 320 °C; probe heater temperature, 350 °C; S-Lens RF Level, 50.

### 2.4. Scanning Electron Microscopy

To further judge the damage to vaginal epithelial cells, we used scanning electron microscopy to observe the morphology of the cells. The cell crawls attached in the 12-well plates were washed with PBS, then added with electron microscope fixative (2.5% glutaraldehyde), fixed at room temperature for 2 h, and stored at 4 °C. The samples were dehydrated with different gradients of ethanol (30, 50, 70, 80, 90, 95, and 100%), dried, and sputtered with gold plating. The scanning electron microscope (JSM-IT700HR, JEOL, Tokyo, Japan) was used to observe and acquire images.

### 2.5. Cellular Supernatant Index Assay

In order to detect the content of glycolysis-related metabolites and LDH in the culture supernatant, we processed the supernatant after centrifugation with the glucose assay kit (glucose oxidase method), pyruvic acid, lactic acid, and LDH assay kits (NJJCBIO, Nanjing, China). The inflammatory factors were detected by human interleukin-6 (IL-6) and human interleukin-8 (IL-8) ELISA kit (4 A Biotech, Beijing, China) according to the instructions. Finally, the absorbance was detected with a microplate reader.

### 2.6. Immunofluorescence Staining

Immunofluorescence assays were used to detect the expression of enzymes involved in glycolysis in vaginal epithelial cells. The samples were washed with PBS and fixed in 4% paraformaldehyde solution for 20 min. The cells were placed in 0.3% Triton X-100 solution for 30 min after washing with PBS and blocked with 5% goat serum at room temperature for 1 h. The cells were incubated in rabbit anti-human LDHA antibody or mouse anti-human PKM2 antibody (1:200; Proteintech Group, Inc., Wuhan, China) overnight at 4 °C, washed with PBS, and incubated in FITC AffiniPure goat anti-rabbit IgG (H + L) secondary antibody (1:50; PUMOKE, Wuhan, China) or Alexa Fluor 594 AffiniPure goat anti-mouse IgG (H + L) secondary antibody (1:50; Yeasen Biotech Co., Ltd., Shanghai, China) for 1 h while protected from light. After washing with PBS, 30 μL of antifade mounting medium with DAPI (Beyotime Biotechnology Co., Ltd., Shanghai, China) was added. The confocal laser scanning microscope (LSM 710, ZEISS, Oberkochen, Germany) was used to observe and acquire images, and fluorescence intensity was measured using Image J 1.5.3.

### 2.7. Statistical Analysis

Thermo Compound Discover 2.0 and Xcalibur were used for initial identification and relative quantification of metabolites; SIMCA 14.1 software was used to perform principal component analysis (PCA), orthogonal partial least squares discriminant analysis (OPLS-DA), and permutation tests; and bioinformatics (https://www.bioinformatics.com.cn/) was used to perform heatmap analysis of metabolites. We used one-way ANOVA and Student’s *t*-test to analyze the experimental data and plotted them with GraphPad Prism 9.5.1. We considered a *p*-value of less than 0.05 a significant difference.

## 3. Results

### 3.1. C. albicans Infection Disrupts the Metabolism of Vaginal Epithelial Cells

**Figure 1:**
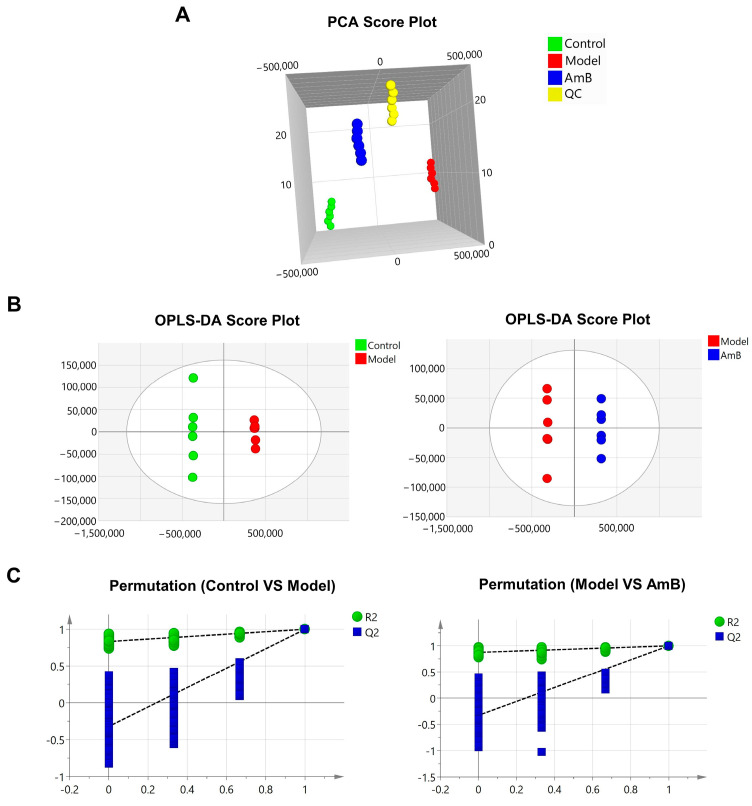
Metabolomics analysis. (**A**) PCA score plot, *n* = 6. (**B**,**C**) OPLS−DA score plot and permutation diagrams (control vs. model; model vs. AmB), *n* = 6.

In order to study the changes in metabolites after *C. albicans* infection of vaginal epithelial cells, we performed metabolomics studies on the cultures. Three groups were set up to explore the disease metabolites of VVC by comparing the model group with the control group, while the target metabolites for the treatment of VVC were studied by comparing the AmB group with the model group. As shown in the PCA plot (Figure 1A) and OPLS-DA (Figure 1B), the metabolic profile data of the control, model, and AmB groups exhibited the characteristics of separation between groups and proximity within groups, which indicated that the difference between groups was obvious, and the experimental methods were stable. The OPLS-DA models were tested for 200 permutations, respectively. The results (Figure 1C) showed that the values of R2 and Q2 on the left side were consistently lower than those on the right side and that Q2 regression lines intersected the negative semiaxis of y, indicating that the models were stable and reliable and did not suffer from overfitting.

**Figure 2:**
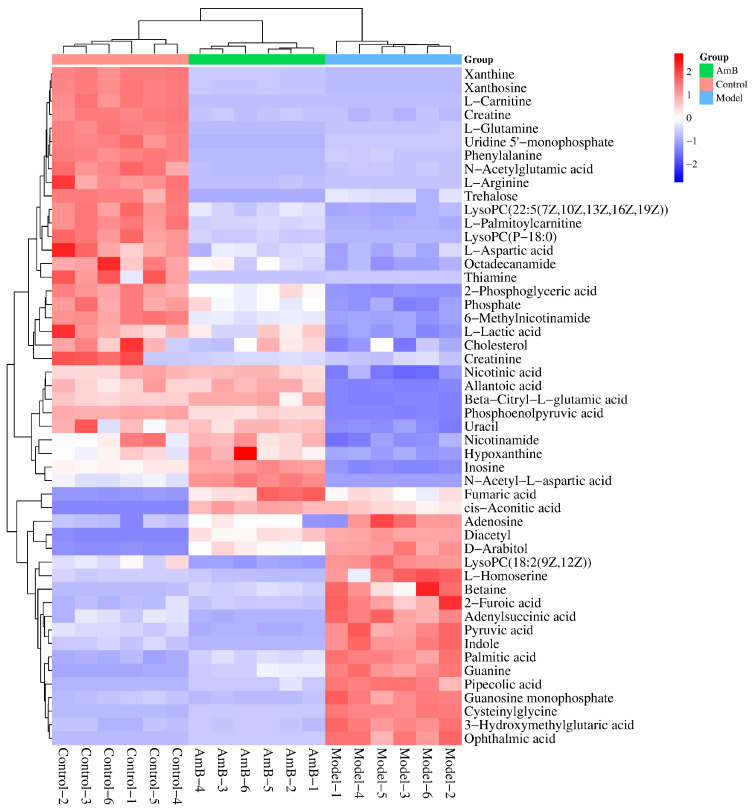
Heatmap of 50 differential metabolites in the model group compared to the control group.

Differential metabolites were screened by setting threshold conditions (fold change value greater than 2 or less than 0.5, *p*-value less than 0.05, variable importance in the projection value greater than 1). As shown in Figure 2, 50 endogenous metabolites were significantly changed in the model group compared to the control group, of which 19 metabolites were up-regulated (D-arabitol, lysoPC(18:2(9z,12z)), betaine, palmitic acid, adenosine, etc.), and 31 metabolites were down-regulated (lactic acid, NAM, NA, phenylalanine, etc.). These metabolites may be potential disease biomarkers for VVC caused by *C. albicans* infection.

### 3.2. Changes in Metabolites after Administration of Antifungal Therapy

Amphotericin B is a polyene antifungal drug commonly used in clinical practice to treat vaginitis, endocarditis, and other fungal infections caused by *C. albicans* infection [17,18]. In this study, amphotericin B was used as an anticandidal-infection strategy to observe whether there is a tendency for the changes in metabolites to return to the control group after controlling the course of the fungal infection and whether the glycolysis of vaginal epithelial cells can return to normal. After administration of AmB for antifungal treatment, 28 metabolites (Table 1) tended to revert to the control group, and we considered these 28 reverse-regulated metabolites as potential biomarkers representing effective control of the VVC pathologic process. Of these 28 metabolites, 15 metabolites (lysoPC(18:2(9z,12z)), adenylsuccinic acid, indole, palmitic acid, 2-furoic acid, guanosine monophosphate, betaine, cysteinylglycine, pipecolic acid, guanine, pyruvic acid, ophthalmic acid, D-arabitol, diacetyl, cis-aconitic acid) were positively correlated with *C. albicans* infection, and 13 metabolites (thiamine, NAM, NA, hypoxanthine, N-acetyl-L-aspartic acid, inosine, uracil, allantoic acid, Beta-citryl-L-glutamic acid, lactic acid, 2-phosphoglyceric acid, phosphoenolpyruvic acid, octadecanamide) were negatively correlated with infection status. 

**Figure 3:**
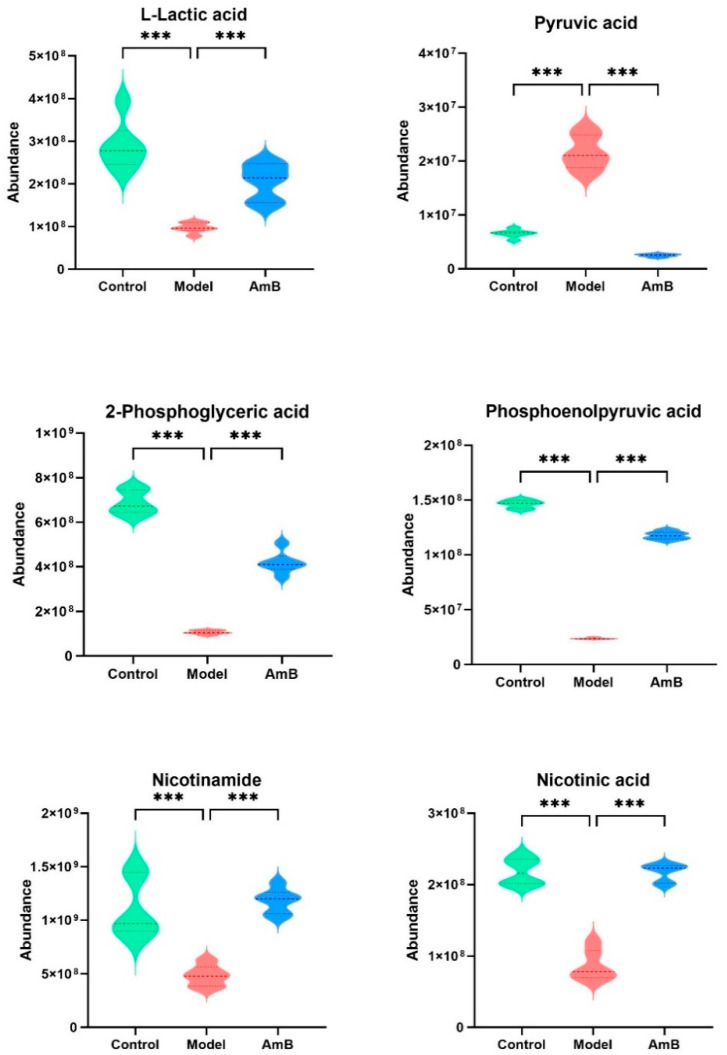
There were significant changes in glycolysis-related metabolites in the model group compared to the control group, and the levels returned to normal after administration. *** *p* < 0.001.

Previous studies have shown that decreased lactic acid was a common feature of reproductive tract infections [19]. In our experiment, the lactic acid content of the model group was significantly reduced, along with significant changes in the levels of pyruvic acid, phosphoenolpyruvic acid, 2-phosphoglyceric, NAM, NA, etc. (Figure 3). The significant changes in the levels of these substances, which were essential metabolites involved in glycolysis, suggested that *C. albicans* infection may cause a disturbance in the glycolysis response of vaginal epithelial cells.

### 3.3. C. albicans Infection Disrupted Glycolysis-Related Metabolites and Enzymes

**Figure 4:**
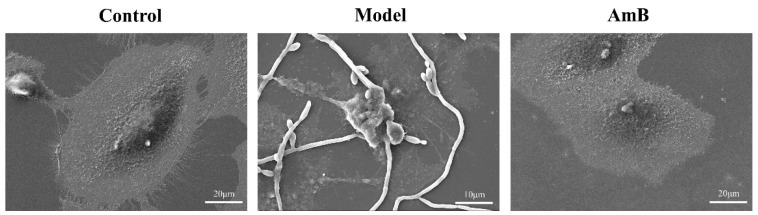
*C. albicans* infection leads to cell membrane damage.

**Figure 5:**
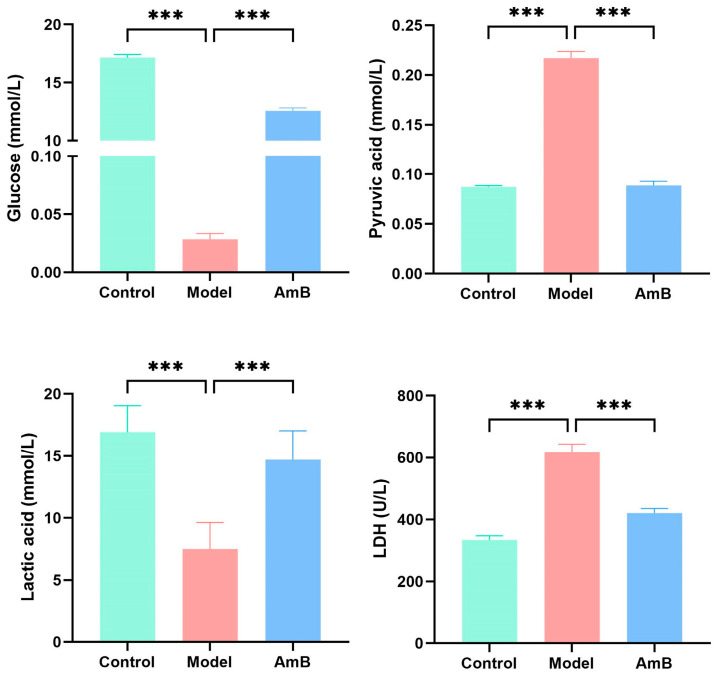
The glycolysis-related metabolites and enzyme (glucose, pyruvic acid, lactic acid, and LDH) levels in the supernatant. *** *p* < 0.001.

To verify the inhibitory effect of *C. albicans* on cellular glycolysis, we observed the damage to cells’ membranes by scanning electron microscopy (Figure 4) and detected the contents of glucose, pyruvic acid, lactic acid, and LDH in the supernatant with a microplate reader (Figure 5). From the scanning electron microscopy results, it can be seen that the cells in the control group had a full and naturally spreading morphology, while the cells in the model group were penetrated and invaded by hyphae, and their surfaces were severely damaged, with ruptured cell membranes, wrinkled morphology, and disappeared pseudopods. In addition, the experimental results showed that the glucose content in the supernatant was significantly reduced compared with the control group. After administration of antifungal therapy, the growth of *C. albicans* was inhibited, and the glucose content was increased considerably. In addition, the pyruvic acid content was elevated, and the lactic acid content was decreased in the model group, which was consistent with the results of metabolomics. LDH, an important metabolic enzyme for glycolysis, as a marker of cell damage, was significantly elevated in the supernatant of the model group, indicating that the cell damage was severe. A large amount of leakage of LDH in the cytoplasm occurred, and the glycolysis of the cells was inhibited.

**Figure 6:**
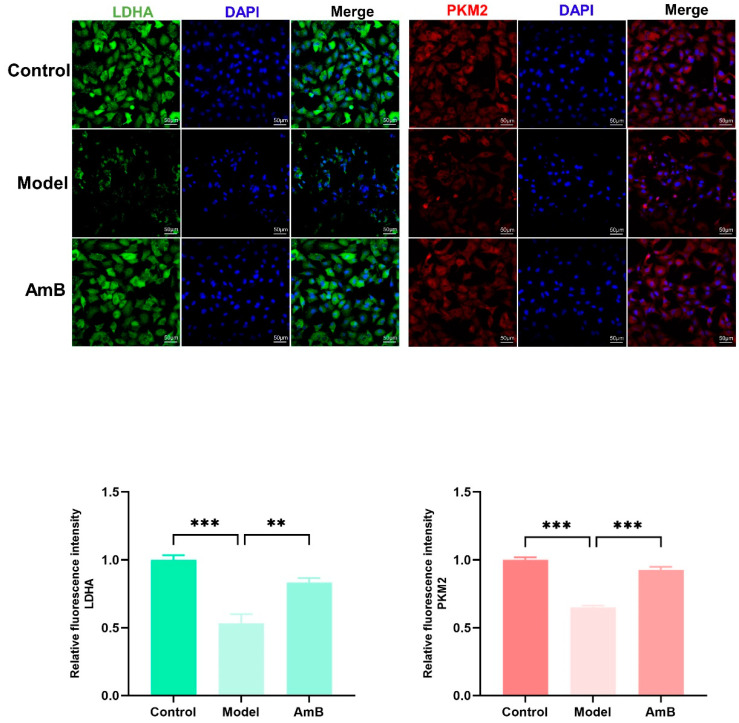
LDHA and PKM2 expression levels in vaginal epithelial cells. ** *p* < 0.01; *** *p* < 0.001.

To further demonstrate the inhibition of glycolysis in vaginal epithelial cells, we examined the key glycolytic metabolizing enzymes, LDHA and PKM2, using immunofluorescence. As shown in Figure 6, the green fluorescence intensity represented the expression degree of LDHA in cells, the red fluorescence intensity represented the expression degree of PKM2 in cells, and the blue fluorescence represented the nucleus. Compared with the control group, the levels of LDHA and PKM2 were significantly lower in the model group, indicating that the glycolysis of infected cells was inhibited; after the administration of antifungal treatment, the levels of these two enzymes were close to those of the control group, indicating that the antifungal treatment restored the cells to the normal level of glycolysis.

## 4. Discussion

Exploring metabolites of diseases is vital for disease prevention, diagnosis, and treatment. Metabolomics, as an important method for detecting endogenous small molecule metabolites, can be used to detect metabolites in body fluids, tissues, and cells for efficient and accurate diagnosis of diseases, and it is often used in the study of various diseases, including cancer, cardiovascular disease, genital inflammation, etc. Gynecological diseases are often closely linked to metabolic disorders, such as cervical cancer, cervicitis, and vaginitis [20,21].

Camilla Ceccarani et al. performed a metabolomic study of vaginal secretions from VVC patients using ^1^H-NMR. They found more than 10 differential metabolites, including lactic acid, phenylalanine, taurine, etc., in samples from VVC patients compared with the healthy group [19]. In our study, we performed metabolomic analysis of an in vitro model of VVC using UHPLC-Q-exact MS, and we found that a total of 50 metabolites were significantly altered in the model group, and these metabolites may be disease biomarkers of VVC. Specifically, we found that lactic acid and phenylalanine levels were also significantly down-regulated in the model group. This further suggested that lactic acid and phenylalanine could be used as disease markers for VVC. In addition, previous studies have shown that D-arabitol is the biomarker for *C. albicans* infection, and GC-MS was used to detect the D-arabitol and L-arabitol ratio in urine to determine the presence of *C. albicans* infection [22]. In our experiments, the D-arabitol level was significantly elevated in the model group and significantly decreased after drug administration, suggesting that D-arabitol can be used as one of the biomarkers for diagnosing VVC.

In addition, these significantly altered potential metabolites we have identified are closely linked to disease. Lysophosphatidylcholine is considered a deleterious pro-inflammatory mediator, with levels elevated considerably when inflammation occurs in the body [23], and has been implicated as a biomarker for cervical cancer [24]. Palmitic acid, a saturated fatty acid, activates toll-like receptors to promote inflammation [25]. Under hypoxic and inflammatory conditions, adenosine levels are significantly elevated to inhibit inflammation [26], but persistently high adenosine levels favor tumor cell growth, promoting cancer [27]. Thiamine is a kind of B vitamin that has an important effect on cellular energy metabolism, and a deficiency can cause a decrease in ATP production and lead to cell death [28]. Betaine (trimethylglycine) is an osmoprotective agent that is anti-inflammatory and regulates energy metabolism [29], but a positive correlation has been reported between betaine concentrations and reproductive inflammation [21]. Maintaining normal levels of amino acids is important for health, and disturbances in amino acid metabolism often accompany genital inflammation. In the present study, significant changes in amino acids were observed in the model group, including phenylalanine, L-homoserine, cysteinylglycine, L-glutamine, L-arginine, N-acetyl-L-aspartic acid, Beta-citryl-L-glutamic acid, and L-aspartic acid. This may be due to the secretion of the virulence factor SAPs by *C. albicans*, which allows the hydrolysis of various proteins in the vaginal epithelium, leading to disturbed amino acid metabolism in the VVC [19].

In particular, we found that metabolites involved in the glycolysis, nicotinate, and nicotinamide metabolism pathways were significantly altered in the VVC model, including 2-phosphoglyceric acid, phosphoenolpyruvic acid, pyruvic acid, lactic acid, NAM, and NA.

**Figure 7:**
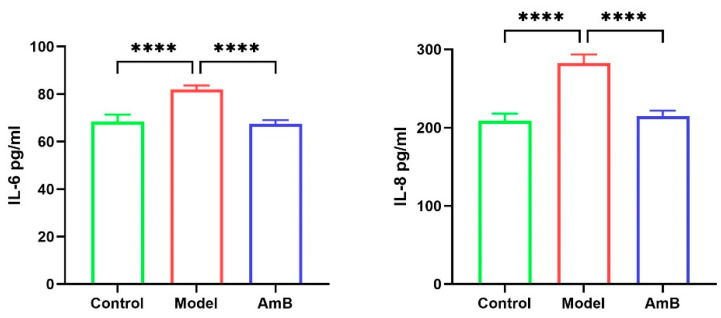
The inflammatory factors’ (IL-6, IL-8) levels in the supernatant. **** *p* < 0.0001.

Lactic acid is essential for maintaining the stability of the intravaginal environment. Lactic acid produced by vaginal epithelial cells and lactic acid produced by *Lactobacillus* maintains the pH of the vaginal environment against the growth of harmful microorganisms [30]. Some studies have shown that lactic acid inhibits the growth of *C. albicans* [31]. The innate immune mechanism of vaginal epithelial cells plays a crucial role in antibacterial infections [32]. Notably, lactic acid can further modulate local immunity within the vagina by stimulating the production of anti-inflammatory factors by human vaginal epithelial cells and inhibiting the production of inflammatory factors (IL-6 and IL-8) triggered by toll-like receptors [33]. This was corroborated by the ELISA results (Figure 7), which showed that the levels of the inflammatory factors IL-6 and IL-8 in the cell supernatants were significantly lower in the control and AmB groups, which had normal lactic acid levels, than in the model group, which had lower lactic acid levels.

**Figure 8:**
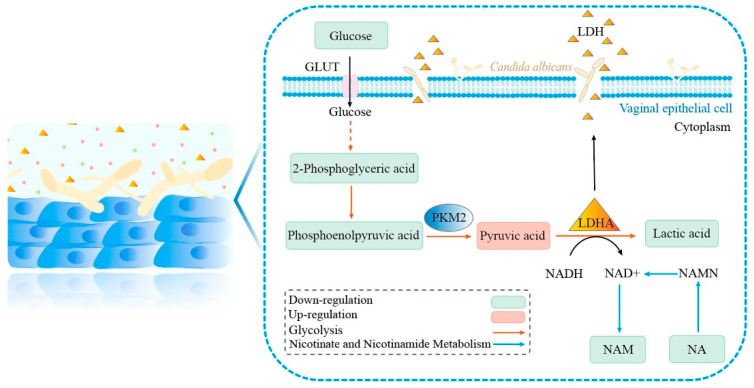
Schematic representation of the metabolic effects of *C. albicans* on vaginal epithelial cells. The green borders represent down-regulation of metabolite levels and red borders represent up-regulation of metabolite levels; red arrows represent the glycolysis and blue arrows represent the nicotinate and nicotinamide metabolism.

As shown in Figure 8, in the hypoxic vaginal cavity, vaginal epithelial cells can produce ATP by anaerobic glycolysis and produce lactic acid to diffuse into the vaginal cavity. Briefly, glucose is metabolized to a series of glycolytic intermediates such as glucose-6-phosphate, glyceraldehyde 3-phosphate, 2-phosphoglyceric acid, etc. 2-phosphoglyceric acid is converted to phosphoenolpyruvic acid, which PKM2 catalyzes to produce pyruvic acid, and eventually pyruvic acid is catalyzed by LDH to produce lactic acid [15,33]. Compared with the control group, the glucose content in the supernatant of the model group was significantly lower, which implied that *C. albicans* infection depleted a large amount of glucose in the culture system, which may lead to a decrease in the amount of glucose that enters vaginal epithelial cells to participate in glycolysis. In addition, after the cells were attacked by *C. albicans*, the expression of LDHA and PKM2 was inhibited, which prevented normal glycolysis in the cells. Thus, the production of metabolites of glycolysis was reduced. The metabolomics results showed a significant decrease in 2-phosphoglyceric acid, phosphoenolpyruvic acid, and lactic acid and an increase in pyruvic acid in the model group, which was consistent with the results of pyruvic acid and lactic acid detection in the culture medium. The increased pyruvic acid content in the model group may be due to the accumulation of pyruvic acid as a result of the significant reduction in LDH preventing its normal conversion to lactic acid.

NADH is oxidized to nicotinamide adenine dinucleotide (NAD+) during the LDHA-catalyzed conversion of pyruvic acid to lactic acid [34]. NAD+ is an essential coenzyme in cellular metabolism and is indispensable for glycolysis. NAM and NA are both precursors of NAD+, producing NAD+ to maintain cellular homeostasis. NAM and NA are both B vitamins necessary for living cells, and NA deficiency can lead to mucosal inflammation [35]. NAM and glycolysis are closely linked. As an inhibitor of PARP-1, it avoids the blocking effect of glycolysis caused by PARP-1. In addition, glycolysis produces NAD+, which produces NAM in the presence of enzymes [36]. NAM has been shown to reduce oxidative stress and has anti-inflammatory effects [37]. NAM is reported to have significant anti-*C. albicans* activity and can synergize with amphotericin B to play a common antifungal role [38,39]. In our study, the levels of NAM and NA were significantly lower in the model group, which may be attributed to the reduced expression of LDHA in the cells and the inhibition of anaerobic glycolysis, reducing NADH conversion to NAD+.

It can be seen that metabolites such as lactic acid, NAM, and NA, which are closely related to glycolysis, are essential for maintaining the health of the human intravaginal environment and that *C. albicans* infection causes disruptions in cellular glycolysis, which disrupts the balance of these natural protective factors, allowing the body’s biological protective effects to be adversely affected.

## 5. Conclusions

In this study, we revealed the metabolic disorders and inhibition of glycolysis in vaginal epithelial cells induced by *C. albicans* infection through metabolomics and biological validation, which provided a reference for the clinical diagnosis and treatment of VVC caused by *C. albicans* infection. In addition, our findings provide new insight for further exploration of *C. albicans* pathogenesis.

## Figures and Tables

**Table 1:** Significant reversal of regulation of 28 differential metabolites after administration of antifungal therapy.( The “↑” symbol represents an increase in metabolite levels and the “↓” symbol represents a decrease in metabolite levels.)

No.	Name	HMDB	Formula	*m*/*z*	RT (Min)	Model vs. Control	AmB vs. Model
1	LysoPC (18:2(9Z,12Z))	HMDB0010386	C_26_H_50_NO_7_P	519.33	10.71	↑	↓
2	Adenylsuccinic acid	HMDB0000536	C_14_H_18_N_5_O_11_P	463.07	2.74	↑	↓
3	Indole	HMDB0000738	C_8_H_7_N	117.06	5.67	↑	↓
4	Palmitic acid	HMDB0000220	C_16_H_32_O_2_	273.27	8.91	↑	↓
5	2-Furoic acid	HMDB0000617	C_5_H_4_O_3_	112.01	1.47	↑	↓
6	Guanosine monophosphate	HMDB0001397	C_10_H_14_N_5_O_8_P	381.07	1.69	↑	↓
7	Betaine	HMDB0000043	C_5_H_11_NO_2_	117.08	1.06	↑	↓
8	Cysteinylglycine	HMDB0000078	C_5_H_10_N_2_O_3_S	161.01	1.45	↑	↓
9	Pipecolic acid	HMDB0000070	C_6_H_11_NO_2_	147.09	1.45	↑	↓
10	Guanine	HMDB0000132	C_5_H_5_N_5_O	134.02	1.69	↑	↓
11	Pyruvic acid	HMDB0000243	C_3_H_4_O_3_	106.03	1.53	↑	↓
12	Ophthalmic acid	HMDB0005765	C_11_H_19_N_3_O_6_	289.13	1.51	↑	↓
13	D-Arabitol	HMDB0000568	C_5_H_12_O_5_	152.07	1.02	↑	↓
14	Diacetyl	HMDB0003407	C_4_H_6_O_2_	104.05	1.49	↑	↓
15	cis-Aconitic acid	HMDB0000072	C_6_H_6_O_6_	174.02	1.08	↑	↓
16	Thiamine	HMDB0000235	C_12_H_16_N_4_OS	264.10	1.22	↓	↑
17	Nicotinamide	HMDB0001406	C_6_H_6_N_2_O	122.05	1.43	↓	↑
18	Hypoxanthine	HMDB0000157	C_5_H_4_N_4_O	136.04	1.43	↓	↑
19	N-Acetyl-L-aspartic acid	HMDB0000812	C_6_H_9_NO_5_	175.05	1.12	↓	↑
20	Inosine	HMDB0000195	C_10_H_12_N_4_O_5_	268.08	1.70	↓	↑
21	Uracil	HMDB0000300	C_4_H_4_N_2_O_2_	95.00	1.50	↓	↑
22	Nicotinic acid	HMDB0001488	C_6_H_5_NO_2_	123.03	1.18	↓	↑
23	Allantoic acid	HMDB0001209	C_4_H_8_N_4_O_4_	176.05	0.92	↓	↑
24	Beta-Citryl-L-glutamic acid	HMDB0013220	C_11_H_15_NO_10_	321.07	1.60	↓	↑
25	L-Lactic acid	HMDB0000190	C_3_H_6_O_3_	90.03	1.43	↓	↑
26	2-Phosphoglyceric acid	HMDB0000362	C_3_H_7_O_7_P	185.99	1.26	↓	↑
27	Phosphoenolpyruvic acid	HMDB0000263	C_3_H_5_O_6_P	167.98	1.39	↓	↑
28	Octadecanamide	HMDB0034146	C_18_H_37_NO	283.29	14.69	↓	↑

## Data Availability

The data presented in this study are available upon request from the corresponding author.
